# Congenital Fibrosis of Extraocular Muscles: A Retrospective Study of 76 Patients

**DOI:** 10.7759/cureus.61909

**Published:** 2024-06-07

**Authors:** Elmas Yuksel Sukun, Aslı Hamis Inal, Osman Bulut Ocak, Mehmet Ozveren, Birsen Gökyigit

**Affiliations:** 1 Department of Ophthalmology, Alaaddin Keykubat University Alanya Education and Research Hospital, Antalya, TUR; 2 Department of Ophthalmology, Beyoglu Eye Training and Research Hospital, Istanbul, TUR; 3 Department of Ophthalmology, Edirne Sultan 1. Murat State Hospital, Edirne, TUR; 4 Department of Ophthalmology, Yeni Yüzyıl University Medical Faculty Eye Clinic, Istanbul, TUR

**Keywords:** congenital, fibrosis, head position, surgery, ocular alignment, strabismus

## Abstract

Background

Congenital fibrosis of the extraocular muscles (CFEOM) is a non-progressive sporadic or familial disease characterized by abnormal innervation of the extraocular muscles. This study aims to evaluate the types of diseases, management steps, and surgical outcomes.

Methodology

A total of 76 patients diagnosed with CFEOM between 2000 and 2022 were evaluated retrospectively. Patients were divided into CFEOM 1, 2, or 3 based on clinical findings. Preoperative and postoperative ocular deviations, as well as abnormal head positions (AHPs), of the patients were evaluated. Excellent outcomes for the head position were defined as less than 5°, good as less than 10°, and poor as greater than 10°. Excellent alignment for strabismus was considered to be less than 10 prism diopters (PD), good as less than 20 PD, and poor as greater than 20 PD.

Results

The average age at the first surgery in our clinic was 11.6 (1-51) years. The mean follow-up was 28.6 ± 7.4 months (range = 4-56 months). Type 1 disease was detected in 48 (63.2%) patients, type 2 disease in eight (10.5%), and type 3 disease in 20 (26.3%) patients. Of the 49 patients with AHP, 20 achieved excellent outcomes, 17 had good outcomes, and the remaining had poor outcomes. Ocular alignment in the primary position following the latest surgery was excellent in 30 patients, good in 26 patients, and poor in 20 patients.

Conclusions

No single best surgical method can be universally applied to every patient diagnosed with CFEOM. Patients must be evaluated individually and carefully, and the appropriate surgical procedure must be chosen. In this way, satisfactory gaze alignment and improvement of the AHP can be achieved.

## Introduction

Congenital fibrosis of the extraocular muscles (CFEOM) is a condition characterized by restrictive ophthalmoplegia, bilateral ptosis, and partial or complete downward fixation of the eyes. The disease is characterized by abnormal residual eye movements, and its genetic inheritance is autosomal dominant or recessive. The clinical appearance is thought to result from myopathic fibrosis of the muscles. The absence of alpha motor neurons in the superior portion of the oculomotor nerve is associated with abnormalities in the levator palpebrae superioris and superior rectus muscles. The presence of central mitochondrial clusters and an increased number of inner nuclei in other extraocular muscles suggests that this pathology also affects other muscles innervated by the oculomotor nerve [1]. Congenital blepharoptosis, head tilt, chin elevation, and primary gaze fixed in hypo- and exotropic positions have been reported in families with CFEOM. The diagnosis is confirmed by a positive forced duction test in the affected eye. Individuals may exhibit similar symptoms, but they may experience varying degrees of restriction in eyeball movements [2]. CFEOM is a heterogeneous group of disorders that may be associated with other anomalies. Genetic inheritance is autosomal dominant in cases reported to be related to chromosome 12, whereas autosomal recessive inheritance has been reported in cases associated with ulnar hand anomalies. Ophthalmologic involvement has been associated with oligodactyly or oligosyndactyly of the hands in patients with non-progressive restrictive ophthalmoplegia and blepharoptosis in the right eye. A genomic scan mapped CFEOM/U to a 1.5 Mb region on chromosome 21qter [3]. Characterized by congenital, non-progressive, restrictive ophthalmoplegia and ptosis, congenital extraocular muscle fibrosis is an autosomal dominant disorder caused by mutations of the *KIF21A* gene. Mutational analysis of the *KIF21A* gene should be performed in cases with typical ophthalmologic findings and positive family history to provide diagnosis, prognosis, genetic counseling, and the possibility of prenatal diagnosis [4]. Congenital orbital fibrosis is a distinct clinical entity characterized by the presence of a non-familial, unilateral, diffusely infiltrating orbital mass [5]. In congenital orbital fibrosis, the oculomotor and abducens nerves are normal, and an infiltrating retrobulbar mass between the extraocular muscles is distinctive from CFEOM [6].

MRI may show hypoplasia of the cranial nerves and support the diagnosis of CFEOM in cases of complicated strabismus. In the clinical evaluation of CFEOM, myosis is a rare condition that may be confused with Horner’s syndrome, characterized by ptosis and miosis, and may lead to misdiagnosis [7].

When examining the causes of paralytic strabismus in children, this condition is rare, affecting approximately 0.1% of children. Ocular motor paresis is characterized by fourth nerve palsy in 36%, sixth nerve palsy in 33%, third nerve palsy in 22%, and multiple ocular motor nerve palsies in 9% of cases. Tumors are rare in children with fourth cranial nerve palsies and are usually associated with other neurological disorders. Rare causes of external ophthalmoplegia that should be considered clinically when the mobility pattern is variable or does not match the ocular motor nerve pattern include CFEOM and myasthenia gravis. Myasthenia often presents as ptosis with exotropia [8]. This study aims to evaluate the types of diseases, management procedures, and surgical outcomes.

## Materials and methods

This study was approved by the Clinical Research and Ethics Committee on December 6, 2016 (approval number: 560). The study was performed in accordance with the Declaration of Helsinki. In this retrospective observational study, patient data were retrieved from hospital archives. Consent forms were obtained from all patients for the surgical procedures performed. Hence, a consent form was not obtained from the patients for the study.

In our study, 76 patients diagnosed with CFEOM between March 2000 and March 2022 were retrospectively analyzed. The patients were evaluated in terms of demographic characteristics, distant and near deviations, and gaze constraints. Refraction, visual acuity, presence of a squint and its pattern, and abnormal head position were recorded. Whether patients had previously undergone ptosis or strabismus surgery, the number of surgeries, and the surgical and clinical success of the procedures were also documented. The family history of the patients was investigated, and CFEOM was classified into types 1, 2, and 3 based on clinical findings.

Even though the diagnosis of congenital extraocular muscle fibrosis syndrome is sometimes confirmed through radiographic and genetic methods, it is typically based on clinical findings. Although we made the preliminary diagnosis based on our clinical examination of the patients, this diagnosis was confirmed by the forced traction test performed on the operating table. In the forced traction test, the exaggerated traction and rotation method described by Guyton was applied. It was observed that the orbital connective tissue lost its elasticity when surgical dissection of the muscles was performed during the operation.

All patients diagnosed with CFEOM in our clinic were included in the study. The gaze constraint data were collected by averaging the constraint gap values of both eyes in the relevant direction. In patients with unilateral disease, only the data from the eye affected by the disease was included in the calculation. In the visual acuity comparisons, the average of the best-corrected visual acuity values of the two eyes was used. The data of the eye with the disease was included in the calculation for patients with unilateral disease. Vertical Krimsky values reflected vertical shifts, while horizontal Krimsky values reflected horizontal shifts. All parameters were created by evaluating the hospital archive records.

Excellent outcomes for the head position were defined as less than 5°, good as less than 10° (5°-9°), and poor as greater than 10°. Excellent alignment for strabismus was considered to be less than 10 prism diopters (PD), good as less than 20 PD (10-19 PD), and poor as greater than 20 PD.

Statistical analysis

The SPSS Statistics version 25 (IBM Corp., Armonk, NY, USA) was used for the statistical analysis of the study. Descriptive statistics were conducted on the data, and the normal distribution was initially assessed. Upon normal distribution of the data, the paired t-test was applied to determine postoperative outcomes. A p-value <0.05 was considered statistically significant.

## Results

The mean age of the patients included in the study was 28.6 ± 19.9 years (minimum = 1, maximum = 51). The mean age at first surgery was 11.6 ± 5.9 years (minimum = 1, maximum = 51). The most common disease pattern was type 1 (n = 48, 63.2%), followed by type 3 (n = 20, 26.3%), and type 2 (n = 8, 10.5%). Patients underwent a mean of 1.9 ± 1.1 (range = 1-5) operations and were followed up for a mean of 28.6 ± 7.4 months (range = 4-56). When the family history of this disease was questioned, 25 out of 76 patients (32.8%) had a positive family history (Table 1).

**Table 1:** Descriptive characteristics of congenital fibrosis of the extraocular muscles patients.

Variables	Mean + SD (minimum-maximum) or n (%)
Age	28.6 ± 19.9 (minimum = 1, maximum = 51)
Age at the first surgery	11.6 ± 5.9 (minimum = 1, maximum = 51)
Gender	
Male	38 (50%)
Female	38 (50%)
Disease type	
Type 1	48 (63.2%)
Type 2	8 (10.5%)
Type 3	20 (26.3%)
Mean number of operations	1.9 ± 1.1 (1-5)
Mean follow-up	28.6 ± 7.4 months (4-56)
Family history	25 (32.8%)

While nine (11.8%) patients had astigmatism greater than 3 diopters in at least one eye, 17 (22.3%) patients had astigmatism between 1 and 3 diopters. Myopia was detected in 47 (61.8 %) patients, while 29 (38.2%) patients were found to have hyperopia. The average age in myopic patients was 35.6 ± 17.7, while it was 25.4 ± 20.4 in non-myopic patients.

The best-corrected visual acuity was 0.60 ± 0.34 in the right eye and 0.49 ± 0.35 in the left eye, as determined by the Snellen chart. In this data analysis, only the data from eyes with the disease were included in the calculation. As a result, amblyopia was detected in 62.9% of the right eyes and 77.4% of the left eyes. The mean corrected visual acuity was 0.76 ± 0.23 in patients with orthophoria according to the Snellen chart, while it was 0.46 ± 0.33 in patients with deviation.

Table 2 demonstrates a significant improvement in patients following the operation. Abnormal head position and ptosis are presented in Table 3. Type 1 disease was detected in 40 out of 49 patients with an abnormal head position. Ptosis was found most frequently in type 1 CFEOM patients.

**Table 2:** Postoperative outcomes of congenital fibrosis of the extraocular muscles patients.

Patient number	Preoperative (diopters)	Postoperative (diopters)	Mean difference	95% confidence interval of the difference (lower-upper)	t	P-value
Exotropia (n = 41)						
Distant Krimsky	34.63 ± 12.74	15.32 ± 12.21	19.317	15.347-23.287	9.835	<0.001
Near Krimsky	33.95 ± 12.74	15.15 ± 11.96	18.805	15.012-22.598	10.020	<0.001
Esotropia (n = 12)						
Distant Krimsky	32.92 ± 9.16	14.67 ± 6.56	18.250	14.594-21.906	10.986	<0.001
Near Krimsky	32.08 ± 9.68	10.67 ± 8.32	21.417	17.787-25.046	12.988	<0.001
Hypotropia (n = 15)						
Distant Krimsky	22.73 ± 8.53	13.07 ± 8.31	9.667	6.406-12.927	6.359	<0.001
Near Krimsky	21.20 ± 6.80	12.60 ± 7.18	8.600	5.641-11.559	6.234	<0.001
Hypertropia (n = 8)						
Distant Krimsky	22.63 ± 10.44	10.50 ± 6.82	12.125	6.848-17.402	5.433	<0.001
Near Krimsky	18.40 ± 8.73	9.75 ± 6.27	8.250	4.284-12.216	4.919	<0.001

**Table 3:** Classifications of congenital fibrosis of the extraocular muscles patients.

Variables		Type 1 (n = 48)	Type 2 (n = 8)	Type 3 (n = 20)
Age		28.3 ± 20.3	44.8 ± 18.6	22.7 ± 16.6
Upward constraint		2.9 ± 1.3	3.4 ± 1.1	1.7 ± 1.6
Abnormal head position	Positive	40 (52.6%)	4 (5.3%)	5 (6.6%)
	Negative	8 (10.5%)	4 (5.3%)	15 (19.7%)
Ptosis	Positive	36 (47.4%)	6 (7.9%)	8 (10.5%)
	Negative	12 (15.8%)	2 (2.6%)	12 (15.8%)

In the postoperative evaluation of the patients, 30 (39.4%) had excellent results in strabismus alignment, 26 (34.2%) had good results, and 20 (26.3%) had poor results. In patients with abnormal head positions, 20 (41%) had excellent results, 17 (34.6%) had good results, and 12 (24.4%) had poor results (Table 4).

**Table 4:** Outcome classification of congenital fibrosis of the extraocular muscles patients. Alignment for strabismus: Excellent = <10 diopters, good = 10-19 diopters; poor = >20 diopters. Head position: Excellent = <5°, good = 5-9°. poor = >10°.

Variables	Excellent	Good	Poor
Alignment for strabismus	30 (39.4%)	26 (34.2%)	20 (26.3%)
Head position	20 (41%)	17 (34.6%)	12 (24.4%)

The inferior rectus recession operation was one of the most commonly used procedures for treatment. An adjustable or fixation suture was also preferred in the literature. We used fixation sutures in most patients. Some information about the patients who underwent surgery is presented in the Appendices.

## Discussion

In our study, we presented the surgical approach and results in a large series of patients with CFEOM. In the postoperative evaluation, we classified the post-strabismus alignment and assessed the improvement in abnormal head position. We found a success rate of 73.6% in post-strabismus alignment (excellent outcome: 39.4%; good outcome: 34.2%) and 75.6% success in correcting abnormal head positions (excellent outcome: 41%; good outcome: 34.6%). Sener et al. [9] achieved a 70% success rate in aligning with 36.5% excellent results and 34.5% good results in a series of 52 patients. In the abnormal head position, they achieved an 82% success rate, with 45% excellent results and 37% good results. The similarity of the results suggests that a patient-specific approach is appropriate. In a genetically defined cohort of 13 patients with CFEOM, 10 patients had type 1 CFEOM, and three had type 3 CFEOM. The chin lift posture significantly improved from 24° ± 8° preoperatively to 10.0° ± 8° postoperatively (p < 0.001). CFEOM is a complex strabismus disorder that is challenging to treat surgically. Despite an aggressive surgical approach, multiple procedures may be required to achieve the desired surgical effect. Knowledge of the underlying genetic diagnosis may help inform surgical management [10].

In our study, our patients did not undergo genetic diagnostic tests. Patients diagnosed based on clinical findings and family history were confirmed through an intraoperative forced traction test. The surgical decision-making process in treating this disease was evaluated based on phenotype in our study. Khan suggested that genetic evaluation is not effective in surgical intervention, but genetic testing for a CFEOM-like phenotype should be performed before general anesthesia to exclude pathogenic mutations in *RYR1* (OMIM #180901). This gene has recently been linked to congenital ophthalmoplegia, ptosis with facial weakness, and malignant hyperthermia [11].

In a study investigating the genetic basis of retinal changes, autosomal dominant CFEOM was found to be caused by heterozygous missense mutations of *KIF21A* or *TUBB3*. It is unclear whether the dysinnervation extends beyond the oculomotor system in CFEOM patients. Sixteen patients with CFEOM were screened for mutations in *KIF21A*, *TUBB3*, and *TUBB2B* genes. Six patients had marked optic nerve hypoplasia. Disc diameter, rim width, rim area, and peripapillary nerve fiber layer thickness were significantly reduced in patients with CFEOM compared to controls (p < 0.005). Situs inversus of retinal vessels was observed in five patients. This study provides evidence of structural optic nerve and retinal changes in CFEOM and suggests that the phenotype in CFEOM extends beyond the motor nerves [12].

In our study, most patients with type 1 CFEOM underwent surgery. In a study sharing the surgical clinical experience of type 1 CFEOM, abnormal head position improved in seven patients. It was emphasized that passive motility testing under general anesthesia should guide surgery and that bovine pericardial implantation is safe and effective when conventional surgery is not feasible [13].

In our study, most patients underwent recession of the inferior rectus muscle. In pediatric patients with CFEOM, bilateral inferior rectus recession was found to be effective in correcting abnormal head positioning. However, it was noted to lead to postoperative exotropia due to reduced adduction function resulting from inferior rectus recession. Therefore, it is recommended to schedule horizontal strabismus surgery after confirming the outcomes of inferior rectus recession [14].

Genetically, *TUBA1A* variants have been reported as a cause of CFEOM [15]. One study reported that congenital monocular elevation deficiency may result from a *TUBB3* variant of familial MED and may be considered a limited form of CFEOM [16].

The clinical features of type 3 CFEOM3 caused by *TUBB3* mutations vary from mild ptosis and restricted extraocular movement to severe ocular motility issues and central nervous system abnormalities. The possibility of type 3 CFEOM should be considered when there is a congenital abnormality of extraocular muscle movement and a positive family history [17]. A patient with a history of febrile seizures and focal seizures and an abnormal electroencephalography was diagnosed with epilepsy. MRI showed a hypoplastic corpus callosum. Mutation analysis revealed a novel de novo heterozygous variant in the *TUBB3* gene [18]. In our patient group, epilepsy was detected in three patients.

The exact mechanism of CFEOM is still unclear. Complex gene interactions occur at the beginning of the embryological process during the formation of the cranial neuromuscular unit. Failure in this process results in various dysinnervation phenotypes. These diseases, characterized by extraocular muscle paralysis and fibrosis, are thought to be caused by the effects of irregular innervation on orbital tissue development [19]. CFEOM is phenotypically and genotypically heterogeneous. At least seven causal genes and one locus are responsible for five subtypes, designated CFEOM types 1 through 5 [20].

A meta-analysis examining common disease characteristics in patients with CFEOM, such as ocular alignment and head position before and after surgery, revealed a postoperative improvement in horizontal alignment of 25.39 PD unilaterally and 10.99 PD bilaterally. The study found that 28.25% of the participants had postoperative head alignment results below 5°, while 60.64% had results between 5° and 9° [21]. In our study, a 19.3 PD improvement was achieved in individuals with exotropia. The proportion of patients with head alignment below 5° was 41%, and the proportion of patients with head alignment between 5° and 9° was 34.6%.

Our study has limitations as it was retrospective in design, conducted at a single center, and included a limited number of patients. Furthermore, the absence of genetic data from our patients is a significant limitation of our study.

## Conclusions

Our study constitutes one of the largest series in the literature. Although the phenotypic features of this rare congenital syndrome vary, fibrosis is the underlying characteristic in all cases. It is important to address the abnormal head position that occurs as compensation for limited ocular motility, restricted ocular elevation, and ptosis. In our study, we obtained outstanding outcomes, with a 41% success rate in correcting abnormal head positions and a 39.4% success rate in improving gaze alignment. There is no single best surgical method that can be applied to every patient diagnosed with CFEOM. Patients must be evaluated individually and carefully, and the appropriate surgical procedure must be selected. In this way, satisfactory gaze alignment and improvement of the abnormal head posture can be achieved.

## Appendices

**Table 5:** Summary examination of patients undergoing strabismus surgery and surgical procedures applied to these patients. Abnormal head position (AHP) was measured by a goniometer. The primary position of each globe with the abnormal head forced into primary position was marked on a diagram and ductions were recorded in nine diagnostic positions of gaze using a grading system of 0 to 4 (with 0 indicating full motion and 4 unable to pass the midline). R: right; L: left; MR: medial rectus; LR: lateral rectus; IO: inferior oblique; SO: superior oblique; SR: superior rectus; IR: inferior rectus; ET: esotropia; XT: exotropia; hyper t: hypertropia; hypo t: hypotropia; OT: orthotropia

Case number	Preoperative deviations condition	Preoperative horizontal Krimsky (distant/near)	Preoperative vertical Krimsky (distant/near)	The upward gaze restriction (right-left)	The downward gaze restriction (right-left)	The adduction restriction (right-left)	The abduction restriction (right-left)	AHP	Surgeries at our center	Postoperative horizontal Krimsky (distant/near)	Postoperative vertical Krimsky (distant/near)
1	XT	25-20	0-0	3-0	0-0	4-0	0-0	+	1. Right LR 7 mm recession, full muscle supraposition, IR posterior fibers recession 5.5 mm	10-10	0-0
									2. Right MR 5 mm resection + 12 mm Faden to IR		
									3. Left LR 6 mm recession		
									4. Left SR Faden + MR 5.5 mm resection		
									5. Right LR extra recession + SR tendon nasal transposition + SO tenectomy		
2	XT	45-45	0-0	4-4	4-4	3-3	1-1	+	1. Left LR insertion site was sutured with 5.0 dacron to medial wall, peroperative LR and IR were not found, retrobulbar hemorrhage occurred in the upper nasal	10-10	0-0
3	XT	45-45	0-0	4-4	3-3	3-3	3-1	+	1. Right LR 8 mm recession MR 8 mm resection, Left LR 8 mm recession	20-20	0-0
4	XT, Hypo T. (R/L)	35-45	6-6	4-4	0-0	0-4	0-3	+	1. Left LR recession 8.5 mm + half muscle supraposition, lower rectus 8.5 mm recession + full muscle nasal transposition + MR 10 mm folding	0-0	0-0
5	XT, Hyper T	10-10	10-10	4-4	3-3	0-0	0-0	+	1. Bilateral IR recession + full muscle nasal transposition was performed-right and left FD + left IR was found at 7.5 mm and towards the nasal	6-6	6-6
6	XT	45-45	0-0	2-4	0-3	0-0	0-4	+	1. Left LR resection + transposition of LR to MR field with Y split	16-16	0-0
									2. Left MR 8 mm resection		
7	ET, Hyper T. (L/R)	30-30	20-20	0-0	4-4	0-0	4-2	+	1. Bimedial 5 mm recession + full muscle infra position + lower oblique myotomy	10-10	10-10
									2. Left IR 4.5 mm folding + half muscle nasal transposition		
8	XT, Hypo T. (L/R)	45-40	20-20	2-0	0-0	3-0	0-0	+	1. Left MR 5.5 mm resection + full muscle infraposition + LR 8 mm recession + full muscle infraposition	40-40	20-20
9	OT	0-0	0-0	0-3	0-2	0-2	0-3	-	1. Left LR Y split with 5 mm recession + left IO release + left SR recession	0-0	0-0
10	XT	45-45	0-0	0-3	0-3	0-4	0-3	-	1. Left LR Y split 2/3 top and 1/3 bottom transposition to the MR field	0-0	0-0
11	XT	30-30	0-0	0-3	0-3	3-4	0-3	+	1. Left LR 8 mm recession	0-0	0-0
									2. Left MR 6.5-7 mm resection		
12	XT	45-45	0-0	4-4	2-2	4-4	2-2	+	1. Left LR 12 mm recession + MR 6 mm fold	25-25	0-0
13	XT, Hyper T. (L/R)	45-45	30-30	0-1	0-3	0-3	0-2	+	1. Left SR 9 mm (3 mm hang bag suture) recession + LR 8 mm recession	45-45	25-25
									2. Left IO nasal transposition, MR 8 mm folding		
14	XT, Hyper T. (R/L)	45-45	10-10	4-4	4-4	4-4	3-2	+	1. Bilateral LR 8 mm recession	20-20	10-10
									2. Left MR 5 mm resection		
15	XT, Hyper T.(L/R)	30-30	10-10	0-0	0-4	0-3	0-0	-	1. FD was limited, adhesions were opened. No tissue compatible with SO and SR was found	20-20	10-10
									2. Left LR 6 mm hang-back clearance with backward + IR 5 mm folding		
16	XT	45-45	0-0	3-3	0-0	3-3	0-0	+	1. Bilateral LR 8 mm recession, bilateral IR 6 mm recession	20-20	0-0
									2. Left LR 14 mm retraction, left MR 6 mm fold		
									3. Bilateral SR 6 mm center fold, SO desensitization		
17	XT	45-20	0-0	3-3	3-3	0-3	0-4	+	1. Left LR regressed 7 mm IR regressed 4 mm, right IR diagonal adhesion was regressed 4 mm, all 3 muscles were found to be excessively fibrotic	10-10	0-0
									2. Right LR 6 mm recession, half muscle supraposition		
18	XT, Hypo T. (L/R)	10-5	10-10	3-3	3-3	3-3	1-1	+	1. FD was restricted on the right, right IR decreased by 6 mm, left LR decreased by 7 mm, FD on the left was found free	0-0	0-0
19	OT	0-0	0-0	4-4	4-4	4-4	4-4	+	1. Bilateral IR 5mm recession + SO desensitization	0-0	0-0
20	Hyper T.( R/L)	0-0	30-30	0-0	0-3	0-3	0-2	-	1. FD was restricted in all directions, retracted 6 mm with SR suspension	0-0	16-16
21	XT	20-45	0-0	0-0	0-0	3-0	1-0	-	1. FD was free in all directions. Wide bands were found in the right MR area, the muscle was fibrotic in its normal place, right MR 5 mm resection + LR was recessed 5.5 mm	30-30	0-0
									2. Right LR 5U BTX		
22	ET	45-45	0-0	0-0	0-0	0-0	3-4	+	1. Right MR 4 mm recession	0-0	0-0
									2. Left MR 5 mm recession + right LR 7.5-8 mm resection		
23	XT, Hyper T. (R/L)	30-25	20-20	2-3	4-0	0-0	0-0	+	1. FD above restricted, right LR found 10-11 mm behind, 5 mm retracted, full muscle supraposition, MR 3 mm resection and supraposition	0-0	5-5
									2. Bilateral IR 2 U BTX		
24	Hypo T. (R/L)	0-0	20-20	4-4	3-3	4-4	4-4	+	1. Bilateral IR 3.5 mm recession	0-0	0-0
25	XT, Hypo T.	20-20	0-0	4-4	3-3	0-0	0-0	+	1. Left IR + LR 4 mm recession	0-0	0-0
26	XT	30-25	0-0	4-4	0-0	3-3	0-0	+	1. Left LR 8 mm recession	10-10	0-0
27	XT	45-45	0-0	4-4	2-3	4-3	0-0	+	1. Bilateral LR desension + MR 8 mm resection	20-20	0-0
									2. Right LR fixed to 12 mm, SO transposed to MR		
									3. Left LR 12 mm recession + SO nasal transposition		
									4. MR detected in the periosteum, LR completely evacuated		
28	ET, Hypo T.	45-45	25-25	4-2	0-0	0-0	2-0	+	1. Right IR 5.5 mm vein protected recession + MR 14 mm Faden + 4 mm recession	18-18	5-5
									2. Left SR brought 5 mm inward and 8 mm back		
									3. Left MR 2.5 mm recession, half muscle supraposition (upper end 4.5 mm, lower end 2.5 mm) + upper margin 13 mm Faden		
29	OT	0-0	0-0	4-4	0-0	0-0	0-0	+	1. Bilateral LR with 7 mm supraposition, IR 4 mm recession + bilateral frontal suspension	0-0	0-0
30	XT, Hyper T. (L/R)	45-45	25-25	4-3	3-2	4-4	0-0	+	1. Left LR 12 mm recession and conjunctiva 8 mm recession, right LR 12 mm recession, right IO 5 mm recession	10-10	8-8
									2. Left SR 5 mm recession		
									3. Left LR 3U BTX		
31	XT	30-30	0-0	3-3	0-0	0-0	0-0	+	1. Bilateral LR 7 mm recession, MR Faden, bilateral frontal suspension	10-10	0-0
									2. Left LR recession 5 mm from the original insertion		
32	ET	30-10	0-0	3-2	3-2	2-2	0-0	+	1. Left LR 7 mm resection	8-8	0-0
